# Outcome measures for airway clearance techniques in children with chronic obstructive lung diseases: a systematic review

**DOI:** 10.1186/s12931-020-01484-z

**Published:** 2020-08-17

**Authors:** Eline Lauwers, Kris Ides, Kim Van Hoorenbeeck, Stijn Verhulst

**Affiliations:** 1 Laboratory of Experimental Medicine and Pediatrics, Faculty of Medicine and Health Sciences, University of Antwerp, Universiteitsplein 1, 2160 Wilrijk, Antwerp, Belgium; 2 Infla-Med Research Consortium of Excellence, University of Antwerp, Antwerp, Belgium; 3 Department of Pediatrics, Antwerp University Hospital, Edegem, Belgium

**Keywords:** Airway clearance technique, Respiratory physiotherapy, Pediatrics, Obstructive lung disease, Outcome measures

## Abstract

**Background:**

Airway clearance techniques (ACTs) are an important aspect of the treatment of children with chronic obstructive lung diseases. Unfortunately, a sound evidence base is lacking and airway clearance strategies are largely based on clinical expertise. One of the reasons for the limited evidence is the lack of appropriate outcome measures specifically related to the effectiveness of ACTs. This review discusses all outcome measures applied in previous research in the pediatric population to provide a baseline for future studies.

**Data sources:**

A systematic literature search was performed in PubMed, Web of Science and EMBASE databases. Search terms included chronic obstructive lung diseases and ACTs.

**Study selection:**

Studies were independently selected by the investigators according to the eligibility criteria. After screening, 49 articles remained for further analysis.

**Results and conclusions:**

Data are summarized according to the type of outcome measure. 48 (98%) studies performed pulmonary function tests, 19 (39%) assessed expectorated sputum, 10 (20%) parameters related to disease exacerbation, 8 (16%) oxygenation, 8 (16%) patient-reported outcomes, 5 (10%) exercise capacity and 5 (10%) applied imaging techniques. The synthesis of results showed a high discrepancy between studies due to differences in study design, population and the application of techniques. Since no ‘gold standard’ method could be identified, a combination of different outcome measures is recommended to gain a better understanding and to identify the potential effects of ACTs. An overview of important considerations has been provided to assist researchers in their choice of outcomes in future studies.

## Introduction

Physiotherapy focusing on augmenting mucociliary clearance and facilitating expectoration of excessive mucus is widely prescribed in chronic obstructive respiratory diseases [1–3]. The main goals of airway clearance techniques (ACTs) are to enhance mucus mobilization, reduce airway resistance, improve ventilation and gas exchange and to reduce work of breathing. Although ACTs are an important part of the treatment of our pediatric respiratory population, a sound evidence base is lacking and airway clearance strategies are largely based on clinical expertise [4]. One of the barriers is the lack of adequate outcome measures specifically related to respiratory physiotherapy. In order to evaluate the effectiveness of the therapy, it is necessary to have reliable and valid outcome measures that have sufficient discriminative capability [5–7].

A clinical endpoint in research has been defined as a characteristic or variable that reflects how a patient feels, functions, or survives [8]. For example: pulmonary exacerbations, intravenous antibiotic usage, hospitalizations, quality of life and ultimately survival. When it is not feasible to directly measure the clinical impact, surrogate endpoints are used to establish therapeutic efficacy and to predict clinical benefit. For pulmonary diseases, the accepted surrogate endpoints for drug development by the European Medicines Agency (EMA) are currently lung function parameters and imaging. Unfortunately, for studies evaluating the effectiveness of airway clearance related interventions, a number of these clinical and surrogate endpoints are difficult to evaluate, as most studies only investigate short-term effects and the use of a control group (not receiving any physiotherapy) in a long-term trial is not accepted in most countries. A wide variety of outcome measures have been used in previous studies, but it is not clear which outcomes are appropriate to measure the acute and/or long-term effects related to airway clearance.

This systematic review provides an overview of all outcomes that have been used to evaluate ACTs in the pediatric population and whether or not significant changes were found. Outcome measures suitable for pediatrics require a specific approach. Besides behavioral challenges, the respiratory disease will often be in an early stage, which means sufficient sensitivity of the outcome is needed to detect any treatment effects. The aim of this review is to assist researchers in their choice of outcome measures by considering the qualities and limitations of the techniques. In addition, recommendations for future research to validate and develop new tools are provided, considering that no ‘gold standard’ outcome measure related to respiratory physiotherapy is available.

## Methods

The recommendations of the Preferred Reporting Items for Systematic reviews and Meta-Analyses (PRISMA) statement were followed to ensure a systematic approach and minimize bias [9].

### Search strategy and study selection

Eligibility criteria were defined before the start of the literature search. Clinical studies were included if: the effectivity of ACTs was investigated, the study population suffered from chronic obstructive respiratory diseases and the mean age of the participants was < 18 years. Articles not written in English, case reports, retrospective studies, conference proceedings, reviews and other articles not based on original research were excluded.

References for this review were retrieved from PubMed, web of science and EMBASE electronic databases. The last search was run on the 10th of September 2019. Synonyms of following search terms were used to construct the search query: respiratory physiotherapy, airway clearance technique, postural drainage, percussion, forced expiratory technique, autogenic drainage, active cycle of breathing technique, high-frequency chest wall oscillation, positive expiratory pressure, intrapulmonary percussive ventilation or breathing exercise; combined with: obstructive lung disease, cystic fibrosis, bronchiectasis, primary ciliary dyskinesia, asthma or bronchopulmonary dysplasia

To select eligible articles, the titles and abstracts of the search results were screened in a first stage. In a second stage the content of the full texts of the remaining studies were reviewed. Both stages were performed by two researchers and disagreements were resolved by consensus.

### Data collection and synthesis

A data extraction form was used by the investigators to obtain relevant information from the included articles. The following data were extracted: study design, characteristics of the subjects (including sample size, age and pathology), type and duration of the intervention, outcome parameters, significance level and a description of the results. This information is described in the section ‘Results’ and summarized into tables according to the type of outcome measure.

The levels of evidence (LOE) described by the Oxford Centre for Evidence-based Medicine were assigned to each study [10]. Randomized controlled trials (RCTs) and cohort studies are categorized as follows: level 1b - RCT with narrow confidence interval, level 2b – cohort study and low quality RCT, level 4 – low quality cohort study. In addition, the PEDro scale, based on the Delphi List, was used to evaluate the internal validity of the individual clinical trials [11]. Each of the 11 items were either scored with 1 (present) or 0 (not present or not reported). Non-applicable items due to the study design (e.g., similarity of the groups in an uncontrolled trial) were scored with a 0 as well. To sum the overall PEDro score of the article, the first criterion relating to external validity was not included.

## Results

### Study selection

**Fig. 1:**
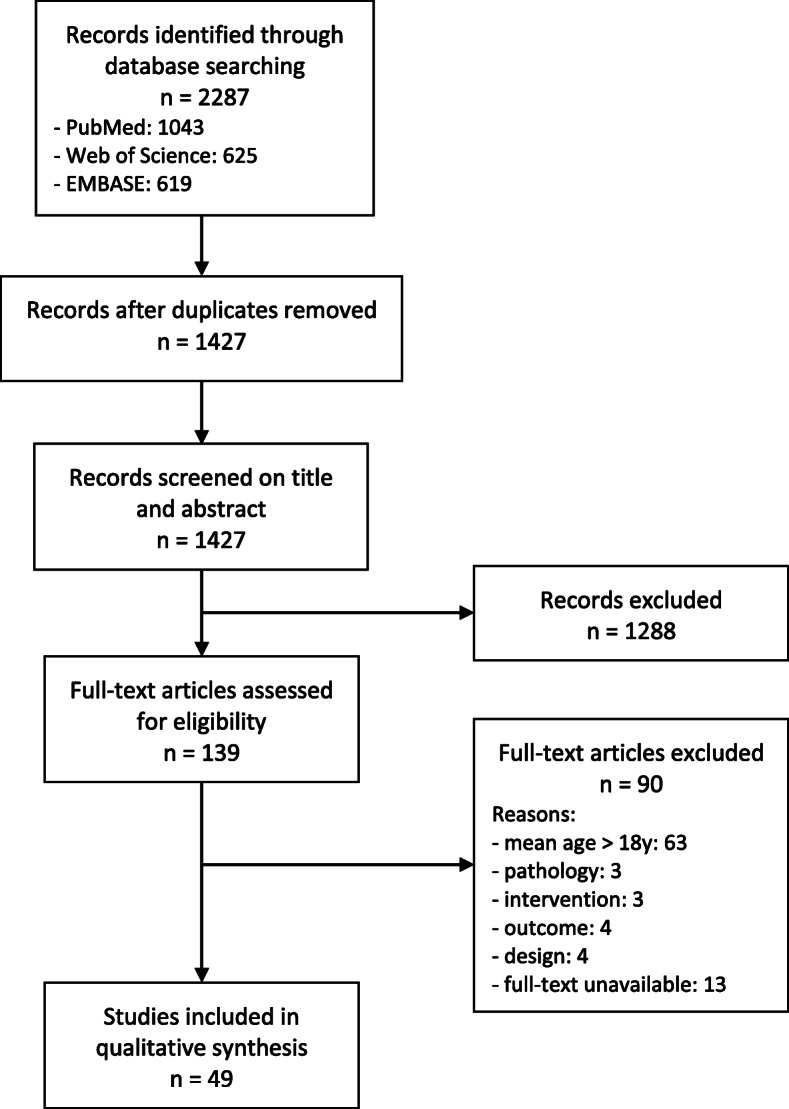
PRISMA flowchart study selection

The database search resulted in a total of 2287 records and after removal of duplicates 1427 remained. Of these, 1288 were discarded after the first screening based on title and abstract. The full texts of the remaining 139 articles were assessed for eligibility, and 90 studies were excluded. This study selection lead to a total of 49 studies, which were all included in the review. An overview of this process is shown in a flow diagram (Fig. 1).

### Study characteristics

Of the 49 articles included in this review, 25 evaluated the acute effects of ACTs after one or more treatment sessions within 24 h [12–36] and 24 evaluated the effects of a longer treatment period in time [37–60]. Twenty-two of these studies were randomized cross-over studies [13–18, 22, 27–30, 32–36, 38, 44, 48, 51, 54, 60], 17 RCTs [19, 20, 31, 39–41, 43, 45–47, 50, 52, 53, 55–58], 7 were uncontrolled prospective trials [21, 23, 25, 26, 42, 49, 59] and three were older studies comparing different treatment sessions in a fixed order in the same group of patients [12, 24, 37]. The majority of the studies (55%) were graded level 2b according to the Oxford LOE [13–16, 19–21, 23, 27, 28, 30–32, 34, 35, 38, 40, 43, 45, 46, 50, 51, 53–55, 57]. Level 1b was assigned to 14 studies (29%) [17, 18, 22, 33, 36, 39, 41, 44, 47, 48, 52, 56, 58, 60] and level 4 to eight studies (16%) [12, 24–26, 37, 42, 49, 59]. There was a great discrepancy between the total number of participants, ranging from 7 to 107 subjects per study. The majority of the studies applied conventional chest physical therapy (CPT), which consisted of postural drainage, percussion and/or vibration, often followed by deep breathing exercises or forced expiration technique (FET) to expectorate sputa [12, 13, 19, 20, 24, 27, 29–31, 34, 35, 37–41, 43, 45, 48, 51, 53–57, 60]. Also, ACTs using devices to facilitate bronchial mucus transport were studied in more than half of the articles, including (oscillatory) positive expiratory pressure ((O)PEP), high-frequency chest wall oscillation (HFCWO) and intrapulmonary percussive ventilation (IPV) [16–18, 23, 27, 32–35, 40–47, 50–52, 54, 59, 60]. Other types of ACTs were breathing techniques (e.g. autogenic drainage (AD), active cycle of breathing technique (ACBT)), exercise [22, 37, 49, 57] and a combination of techniques previously mentioned [14, 15, 25, 26, 28, 29, 33, 36, 58, 60]. Forty-three out of 49 studies included children with cystic fibrosis (CF), of which ten studied children during a respiratory exacerbation [12–19, 21–28, 30–41, 43–46, 48–50, 52, 54–57, 59, 60]. The remaining six articles included children with an acute asthma exacerbation [20, 47, 58], primary ciliary dyskinesia (PCD) [51], human immunodeficiency virus (HIV) [42] and one studied a group of children with different chronic obstructive lung diseases [53]. Although HIV was not included in the search query, the study of Plebani et al. specifically enrolled children with recurrent pulmonary infections requiring prophylactic pharmacological treatment, which corresponds to our population of interest [42]. A summary of the study characteristics can be found in Table 1.

**Table 1:** Study characteristics

Article	Design	Pathology	n	ACT	Duration	Type of outcome measures								LOE
						PFT	SP	OX	EX	IM	DE	PRO	OTH	
Denton 1962 [12]	comparative	CF	23	CPT	1x	•	•							4
Maxwell 1979 [13]	RXO	CF	14	CPT	1x	•	•							2b
Weller 1980 [24]	comparative	CF	20	CPT	2x	•	•							4
Zach 1982 [37]	comparative	CF	10	CPT, exercise	17 d + 8 w	•								4
Desmond 1983 [38]	RXO	CF	10	CPT	3 w	•	•							2b
De Boeck 1984 [30]	RXO	CF	9	CPT	1x	•	•							2b
Andreasson 1987 [49]	uncontrolled	CF	7	Exercise	30 m	•			•	•			•	4
Van Asperen 1987 [54]	RXO	CF	10	CPT, PEP	4 w	•	•					•		2b
Bain 1988 [55]	RCT	CF	38 (19/19)	CPT	2 w	•	•	•						2b
Reisman 1988 [56]	RCT	CF	63 (30/33)	CPT, FET	3 y	•			•		•		•	1b
Cerny 1989 [57]	RCT	CF	17 (8/9)	CPT, exercise	13 d	•	•		•					2b
Maayan 1989 [31]	RCT	CF	25 (6/5/8/6)	CPT	1x	•								2b
Asher 1990 [58]	RCT	Asthma	38 (19/19)	CPT	2 d	•								1b
Oberwaldner 1991 [59]	uncontrolled	CF	18	PEP	16 d	•								4
Steen 1991 [60]	RXO	CF	28	CPT, PEP, FET	4 w	•	•					•		1b
Van der Schans 1991 [32]	RXO	CF	8	PEP	1x	•				•				2b
Pfleger 1992 [33]	RXO	CF	14	PEP, AD	1x	•	•							1b
Bauer 1994 [39]	RCT	CF	73 (36/37)	CPT	12 d	•					•			1b
Natale 1994 [34]	RXO	CF	9	IPV	5 d	•	•							2b
Homnick 1995 [40]	RCT	CF	16 (8/8)	CPT, IPV	180 d	•					•	•		2b
McIlwaine 1997 [41]	RCT	CF	36 (18/18)	CPT, PEP	1 y	•				•	•			1b
Plebani 1997 [42]	Uncontrolled	HIV	8	PEP	1 y	•					•			4
Homnick 1998 [43]	RCT	CF	33 (16/17)	CPT, OPEP	9 d	•					•		•	2b
Newhouse 1998 [35]	RXO	CF	10	CPT, OPEP, IPV	1x	•	•							2b
Van Winden 1998 [44]	RXO	CF	22	PEP, OPEP	2 w	•								1b
Fauroux 1999 [36]	RXO	CF	16	FET, NIV	1x	•	•	•				•		1b
Gondor 1999 [45]	RCT	CF	20 (8/12)	CPT, OPEP	2 w	•			•					2b
Williams 2000 [14]	RXO	CF	26	CPT, ACBT	1x	•	•						•	2b
McIlwaine 2001 [46]	RCT	CF	40 (20/20)	PEP, OPEP	1 y	•				•	•		•	2b
Williams 2001 [15]	RXO	CF	15	CPT, ACBT	1x	•							•	2b
Samransamruajkit 2003 [47]	RCT	Asthma	40 (20/20)	OPEP	2 d	•		•					•	1b
Marks 2004 [27]	RXO	CF	10	CPT, IPV	1x	•	•					•		2b
Phillips 2004 [16]	RXO	CF	10	HFCWO, ACBT	2x	•	•					•		2b
Darbee 2005 [17]	RXO	CF	15	HFCWO, PEP	2x	•		•						1b
Lagerkvist 2006 [18]	RXO	CF	15	PEP, OPEP	1x	•		•						1b
Hristara-Papadopoulou 2007 [29]	RXO	CF	35	CPT, ACBT	1x		•							2b
Indinnimeo 2007 [53]	RCT	multiple	19 (12/7)	CPT	1 m	•								2b
Tannenbaum 2007 [19]	RCT	CF	18 (9/9)	CPT	1x	•								2b
Didario 2009 [20]	RCT	Asthma	40 (20/20)	CPT	6x in 24 h	•		•						2b
Bannier 2010 [21]	Uncontrolled	CF	10	Breathing exercises	1x	•	•			•				2b
McIlwaine 2010 [48]	RXO	CF	18	CPT, AD	1 y	•					•	•	•	1b
Reix 2012 [22]	RXO	CF	32	ACBT, exercise	1x	•	•					•		1b
Abbas 2013 [23]	Uncontrolled	CF	25	PEP/OPEP	1x	•								2b
McIlwaine 2013 [50]	RCT	CF	107 (51/56)	PEP, HFCWO	1 y	•					•	•		2b
Gokdemir 2014 [51]	RXO	PCD	24	CPT, HFCWO	5 d	•		•				•		2b
Hortal 2014 [25]	Uncontrolled	CF	8	AD, PEP	1x	•								4
Voldby 2018 [26]	Uncontrolled	CF	10	Exercise, PEP	2x	•								4
Ghasempour 2019 [52]	RCT	CF	40 (20/20)	PEP	6 d	•		•			•			1b
Vendrusculo 2019 [28]	RXO	CF	12	AD, PEP	1x	•			•					2b

*Abbreviations*: *ACBT* active cycle of breathing technique, *ACT* airway clearance technique, *AD* autogenic drainage, *CF* cystic fibrosis, *CPT* chest physical therapy, *DE* disease exacerbation parameters, *EX* exercise, *FET* forced expiration technique, *HFCWO* high frequency chest wall oscillation, *IM* imaging, *IPV* intrapulmonary percussive ventilation, *LOE* level of evidence, *n* number of subjects, *NIV* non-invasive ventilation, (O) PEP (oscillatory) positive expiratory pressure, *OTH* other type of measures, *OX* oxygenation, *PCD* primary ciliary dyskinesia, *PFT* pulmonary function test, *PRO* patient-reported outcome, *RCT* randomized clinical trial, *RXO* randomized crossover trial, *SP* expectorated sputum

### Risk of bias within studies

Results of the risk of bias assessment showed that 15 (31%) studies scored between zero and three points out of ten [12–14, 21, 23–26, 29, 37, 38, 42, 43, 49, 59], 19 (39%) scored four or five [15, 17, 18, 20, 30–32, 35, 39, 40, 46, 47, 51, 53–57, 60], 14 (29%) scored between six and eight [16, 22, 27, 28, 33, 34, 36, 41, 44, 45, 48, 50, 52, 58] and only one study reached a total score of nine points [19]. Concerning the internal validity, the criteria ‘randomization’ and ‘similarity of groups at baseline’ were satisfied in the majority of the studies (73 and 71% respectively). Also, most studies reported sufficient statistical information to make their results interpretable, which was assessed by the criteria ‘between-group statistical comparison’ (80%) and ‘point/variability measures’ (90%). Blinding of the therapists was not possible due to the type of intervention, but 17 (35%) studies blinded the outcome assessors to minimize bias. An overview of all individual scores can be found in the online supplementary material, Table S1.

### Outcome measures

The synthesis is organized as follows: results are shown per type of outcome measure and are further subdivided depending on the duration of the study. For each type of outcome measure a summary table shows the occurrence of significant results found in one or more parameters compared to baseline, a control treatment and/or a different ACT. These tables are shown in the supplementary material (Tables S2–8).

## Pulmonary function tests

### Description

Spirometry is the most widely used pulmonary function test (PFT) in clinical practice as well as in research. To date, the Forced Expiratory Volume in one second (FEV_1_) is the recommended primary surrogate endpoint by the North American Food and Drug Association (FDA) and the EMA for chronic obstructive lung diseases in the pediatric and adult population. Therefore, FEV_1_ has been the most important outcome in pharmacological studies, but also in studies assessing the effectiveness of respiratory physiotherapy. In addition to spirometry, body plethysmography has regularly been performed to measure static lung volumes and airway resistance [61]. These parameters provide additional valuable information about airway obstruction and hyperinflation. Other PFTs performed in several articles included in this review were inert gas washout tests and respiratory muscle strength tests. For all abovementioned PFTs extensive guidelines and reference equations have been developed, which enables clinicians and researchers to obtain valid and reliable results using commercially available equipment [61–63]. Although the majority of the PFTs can be applied in young children, specialized equipment, modified testing procedures and trained staff are required to ensure sufficient quality of the assessments [64].

### Results: short-term effects within 24 h

Of the 25 studies assessing the acute effects of ACTs, 24 measured lung function [12–28, 30–36]. Spirometry was most frequently performed to measure changes in parameters such as FEV1, forced vital capacity (FVC), peak expiratory flow (PEF) and forced expiratory flow between 25 and 75% of the FVC (FEF_25–75%_). Inconsistent results were found for different spirometric parameters, but overall FEV_1_ was most sensitive to acute changes. Of all 17 studies comparing pre to post measurements, merely six found a significant improvement in one or more parameters [16, 17, 24, 30, 33, 35]. The majority of the studies compared the results of two types of ACTs, but Williams et al. and Reix et al. were the only to show a significant difference between treatment sessions [14, 22]. Three studies compared an ACT to a control period [19, 24, 28], but only Weller et al. could show significant effects related to physiotherapy [24].

Body plethysmography was performed in seven studies to measure static lung volumes [15, 27, 28, 30, 32, 33, 35]. No differences were found compared to baseline, except for the study by Pfleger et al. reporting a significant decrease after PEP and the combination of both PEP and AD. A single study compared ACT to a control period and they found a higher functional residual capacity (FRC) after ACT indicating altered lung dynamics [28]. None of the five articles comparing types of ACTs could find differences in any parameters measured by body plethysmography [15, 27, 30, 33, 35].

Besides spirometry and body plethysmography, seven studies applied other types of PFTs [17, 19, 20, 23, 26, 31, 36]. Three of them performed inert gas washout tests [17, 23, 26], of which two could find a significant improvement in at least one parameter compared to baseline [17, 23]. Fauroux et al. and Didario et al. determined airway resistance using the flow interruption technique and impulse oscillometry, respectively, but no changes were found in either study [20, 36]. Maayan et al. evaluated infants, which required the application of modified PFTs suitable for this age group. No significant changes compared to baseline were found using a thoraco-abdominal squeeze jacket and infant whole body plethysmography [31].

### Results: long-term effects

The duration of the intervention in clinical trials evaluating the effectiveness of multiple treatment sessions ranged from two days to three years. All of these studies performed spirometry, complemented by an additional PFT in some of the studies [37–60]. Eighteen studies compared spirometric parameters post-intervention to baseline values [37, 38, 40, 43–47, 49–57, 59], of which eleven found an improvement [37, 43, 45, 47, 50–53, 55, 57, 59]. Only in four articles, ACT was compared to a ‘no treatment period’ [38, 47, 52, 58], and three of them noted a significant beneficial effect of the physiotherapy treatment on lung function [38, 47, 52]. No difference was found by Asher et al., following children for two days during an acute asthma exacerbation [58]. Most studies compared different types of ACTs, but few of them reported significant benefits of one technique over the other [41, 45, 46, 53, 56]. No association could be found between significant results and the intervention period, sample size or risk of bias score of the study. In contrast to studies assessing the acute effects, the studies evaluating a longer intervention period generally found changes in multiple parameters. FEV_1_, FVC and FEF_25–75%_ were most frequently reported to change over time or differ between groups.

Similar results were found for body plethysmography, namely, the majority of the studies could find a significant improvement in time and none showed significant differences between types of ACTs. A total of seven studies measured static lung volumes, of which four found a decrease in hyperinflation after the intervention period [43, 53, 55, 59]. The only study reporting a difference between two treatment regimens was conducted by Indinnimeo et al. comparing supervised to unsupervised CPT [53]. The decrease in hyperinflation remained significantly lower in the supervised group even after one year following the one month intervention period.

Zach et al. and Andréasson et al. measured static lung volumes using two different techniques, helium dilution and nitrogen multiple breath washout, respectively [37, 49]. Although both of them evaluated exercise to increase airway clearance, only Andréasson et al. found a significant decrease in air-trapping. There was, however, a great discrepancy between the intervention periods, namely 30 months in the study by Andréasson et al. and 17 days by Zach et al.

### Discussion

One of the goals of ACTs is to reduce airway resistance and to improve ventilation, which should result in increased dynamic lung volumes and decreased hyperinflation [5]. Nevertheless, PFTs seem insensitive to the acute effects of airway clearance. Although this has already been described by Van der Schans in 2002 [6], pulmonary function remains the most frequently assessed outcome. In contrast to short-term trials, lung function variables are affected by a longer treatment period. However, these differences in time cannot solely be attributed to respiratory physiotherapy, but they are a result of multiple aspects of the treatment and the evolution of the respiratory status in general. The few trials comparing ACTs to a ‘no treatment’ period noted a clear benefit of physiotherapy, but when different types of treatment were compared little to no differences were found. Overall, no association was found between the reported results and the LOE of the studies.

An important limitation of conventional PFTs, such as spirometry and body plethysmography, is that the respiratory system is regarded as a single unit. This reduces the sensitivity of the techniques and no information can be provided about regional abnormalities and changes. Specifically for the pediatric population, conventional PFTs are associated with additional disadvantages related to the required voluntary effort. In infants, lung function is generally tested during sedation, most often using chloral hydrate, which has been reported to be safe and effective for short procedures in infants. Nevertheless, recommendations need to be followed strictly and trained staff is needed to prevent severe adverse events [65]. PFTs in preschool children are associated with other specific challenges, since they are too old to sedate, but they are not yet able to perform reproducible respiratory maneuvers [64]. Additionally, tests such as preschool spirometry are considered too insensitive to identify early airway disease in this young population [66]. Over the past decade, Multiple-Breath Washout (MBW) testing has been a topic of high-interest to assess pulmonary function in preschoolers. This test is more sensitive to abnormalities in the small airways than spirometry and, therefore, MBW is increasingly used as an outcome measure in research [66]. Despite its potential, no studies evaluating ACTs with MBW in this age group have been performed to date. In older children, ventilation inhomogeneity was only measured by Voldby et al., but no acute effects could be detected [26].

## Expectorated sputum

### Description

Besides PFTs, sputum quantity has been one of the most commonly used outcome measures for respiratory physiotherapy. Although the volume or weight of expectorated sputum would be the most direct measurement of airway clearance, this measurement is not considered valid or reliable, especially in the pediatric population. Besides the inability to collect sputum in young and/or uncooperative children, sputum production can be over- or underestimated. Secretions may be swallowed or they might be contaminated with saliva [5, 6]. In addition, the amount of expectorated sputum is highly variable between days as well as at different time points during one day [6]. Besides sputum quantity, the rheological properties can be evaluated by a microrheometer [67]. While this procedure is able to assess viscoelasticity in a standardized way, no studies could be found describing the validity or reliability.

### Results: short-term effects within 24 h

Thirteen studies compared sputum quantity between two types of therapy [13, 14, 16, 22, 27, 29, 30, 33–36, 54, 60], but only Pfleger et al. and Phillips et al. could find significant differences [16, 33]. Natale et al., also evaluated mucus rheology, but they did not observe a difference in consistency between treatment sessions [34]. Only Pfleger et al. included a control session, and they showed a significant higher amount of expectorated sputum during each type of treatment (AD, PEP, and a combination of both) compared to spontaneous coughing [33].

### Results: long-term effects

Four studies compared the sputum quantity collected during different treatment sessions of a longer intervention period [38, 55, 57, 59]. None of them could find any significant differences between types of ACTs. Bain et al. collected sputum at the beginning and at the end of the hospitalization and they showed a decrease in sputum volume at discharge in both groups receiving either CPT or only directed coughing [55]. Oberwaldner et al. could find significant correlations between sputum quantity and changes in several lung function parameters after PEP therapy [59]. Caution is advised when interpreting these results, since all four studies were published over 25 years ago, scored low on the risk of bias assessment and none of them had a high LOE.

### Discussion

Due to the lack of sufficient validity and reliability, sputum quantity cannot be recommended for research in pediatrics to evaluate the effectiveness of ACTs. Based on the included studies, sputum quantity also lacks discriminative capability to compare different treatment regimens. Apart from sputum quantity, mucus rheology can be determined, which is a feature that influences the efficiency of airway clearance. It has previously been reported that airway oscillations could reduce the viscoelasticity [67], which can be induced by techniques such as OPEP and IPV. Of the studies included in this review, only Natale et al. measured viscoelasticity, but no additional benefit of IPV over CPT was noted [34]. Despite the potential relevance of the outcome, little research concerning mucus rheology related to physiotherapy has been performed, which means no conclusive statements can be made.

## Oxygen measurements

### Description

If ventilation improves or sputum is removed from the lungs, it would be logical to expect an improvement in oxygenation. Therefore, many authors have used blood gas changes as an outcome for airway clearance. Arterial blood gas (ABG) analysis is the reference method to assess oxygen saturation and the partial pressure of oxygen as it directly measures the concentration of arterial gases. Oxygen levels can also be assessed indirectly by pulse oximetry, calculating the percentage of oxygenated hemoglobin derived from the absorption of light. Although pulse oximetry is a simple and reliable measure, the devices have a relatively large margin of error (i.e. a 95% confidence interval of +/− 4%) [5, 68].

### Results: short-term effects within 24 h

Five studies assessed the acute effects on oxygen levels by transcutaneous measurements [17, 18, 20, 36, 51]. Four of them monitored peripheral oxygen saturation (SpO_2_) continuously during treatment and three could find significant differences between types of therapy [17, 18, 36, 51]. In these three studies ACTs involved inspiratory or expiratory positive pressure (PEP, OPEP and NIV) leading to a significant increase of SpO_2_ or prevented desaturation during the therapy session [17, 18, 36]. Other types of treatment, including FET and HFCWO, were associated with significant falls in SpO_2_ [17, 36]. In all studies oxygen levels returned to baseline rapidly after treatment. Gokdemir and colleagues also monitored SpO_2_ continuously, but they did not observe any changes compared to baseline or between types of therapy (i.e. CPT and HFCWO) [51]. The fifth study by Didario et al. compared SpO_2_ in a group of asthmatic children before and after CPT to the saturation in a control group, but they did not find any significant differences pre and post treatment in either group [20].

### Results: long-term effects

A total of three studies assessed the changes in oxygen levels over a longer period in time [47, 52, 55], of which only Ghasempour et al. performed ABG analysis [52]. The latter group of researchers included children with CF hospitalized for an acute exacerbation and they analyzed blood gas samples at hospital admission and discharge. A similar improvement was found between the PEP and control group [52]. Bain et al. studied a group of CF patients admitted to the hospital as well and they showed similar results. Oxygen levels measured by pulse oximetry improved, but no differences were found between the group receiving CPT and the group performing directed coughing only [55]. Samransamruajkit et al. compared OPEP (Flutter) to standard treatment without physiotherapy in children with an acute asthma exacerbation [47]. Although the mean SpO_2_ did not differ between groups, they showed that OPEP might reduce the need of supplemental oxygen over time.

### Discussion

It has been observed that ACTs providing positive pressure could effectively improve oxygen saturation. Short-term changes could be shown in several level 1b and 2b studies, but these changes normalized rapidly after therapy [17, 18, 36]. Studies monitoring oxygen levels during a respiratory exacerbation found an improvement toward the end of hospitalization, but no clear evidence could relate these improvements to an airway clearance regimen [52, 55]. Although ABG analysis is the ‘gold standard’ to measure oxygen levels, this test was only performed in one study [52]. Disadvantages of ABG are the invasive procedure and it only provides information about one specific moment in time. As mentioned in the introduction, oxygen saturation can also be measured transcutaneous by pulse oximetry, which can be performed in an outpatient setting and is suitable for continuous monitoring. Unfortunately, the low accuracy compared to ABG, questions its utility to detect small changes in a research setting.

Specifically for the pediatric population, oxygen saturation might not be an appropriate outcome to measure the effectiveness of an intervention, as most children still have normal saturation levels. On the other hand, this outcome might be of vital importance to guide therapists in their treatments in more severe cases.

## Exercise capacity

### Description

Aerobic fitness in children with chronic lung disease has shown to be a predictor of mortality and morbidity in later years [69]. Different types of tests are available, of which the maximal incremental ergometer test and the Six Minute Walk Test (6MWT) are most often performed. The ‘gold standard’ for exercise testing is the incremental exercise test and has been most extensively described in pediatrics. This test provides detailed information about the physiologic response to exercise and can be used for diagnostic, prognostic and evaluative purposes in medicine [69]. Although numerous age-specific reference values have been proposed, they are based on heterogeneous exercise protocols and few of them are corrected for body size and maturation [70]. The 6MWT requires less set-up and is more feasible for long-term monitoring, but in contrast to the adult population, few studies have performed this test in children with chronic obstructive lung diseases at present [71]. In healthy children the 6MWT has shown to be a valid and reliable test to assess functional exercise capacity [72].

### Results: short-term effects within 24 h

Vendrusculo et al. were the only to study the acute effects of airway clearance on exercise capacity [28]. Twelve children with CF performed an incremental exercise test twice, of which one test was preceded by a session of AD and PEP. No differences were found for variables evaluated at peak exercise, but a significant decrease in minute ventilation and ventilatory equivalents were noted at the subject’s ventilatory threshold [28].

### Results: long-term effects

Andréasson et al. and Reisman et al. evaluated the exercise capacity after 2.5 and three years, respectively [49, 56]. The intervention in the study by Andréasson et al. consisted of daily exercise [49], while the children included by Reisman et al. received either a combination of percussion, vibration and FET or FET only [56]. In both studies the maximal workload remained stable over time. No differences were found between the children performing only FET or receiving the combined treatment.

Two studies evaluated exercise capacity at hospital admission and at discharge during an acute exacerbation [45, 57]. Both Cerny et al. and Gondor et al. found an improvement at the end of the hospitalization, but no differences were found between treatment regimens.

### Discussion

Since only one level 2b study used exercise testing to evaluate the acute effects of ACTs, no definite conclusions can be drawn. The study suggests that CPET could be a valuable measure to assess the physiological responses of ACT during exercise [28], but this should be verified in future studies. Four studies evaluated exercise capacity before and after a longer intervention period, but none of them could find a significant difference between types of ACTs [45, 49, 56, 57]. In general, exercise testing is considered suitable to evaluate a rehabilitation program or a multidisciplinary intervention in a long-term clinical study. This outcome lacks sufficient sensitivity to assess local changes in the lungs directly related to ACTs. Caution is required when interpreting the results of exercise tests in children with chronic lung diseases. At present, no data are available on the minimal clinically important difference for neither CPET nor the 6MWT. When measuring exercise capacity in children, several aspects need to be taken into account, such as appropriate equipment and age-specific reference equations. Furthermore, children need to be able to fully understand instructions to perform the maximal effort required.

## Imaging

### Description

Different imaging techniques are available to visualize the airways and lung parenchyma, of which chest X-rays and computed tomography (CT) are most commonly used in clinical practice. Scoring systems have been developed to grade disease severity and to assess images in a more structured and standardized way. For CF, the Brasfield score has been frequently applied for the evaluation of chest x-rays and has shown to have a good interrater agreement [73]. A more recent study compared the Brasfield score to CT imaging, which is considered the ‘gold standard’ for the evaluation of structural abnormalities of the respiratory system. They concluded that the Brasfield score is highly sensitive in detecting chest CT abnormalities [74].

Besides the evaluation of structural abnormalities, imaging techniques can be used to assess functional characteristics as well. Bronchial mucus transport can be directly measured by inhaling a radioactive aerosol tracer (RAT), but the results are highly variable depending on the particle deposition [6]. Recent studies concerning this topic are lacking. Another technique, hyperpolarized Magnetic Resonance Imaging (MRI), is able to assess ventilation distribution and has shown to be a sensitive technique to detect early lung disease [75].

### Results: short-term effects within 24 h

Van der Schans et al. measured mucus transport by a RAT technique, but they did not observe a significant increase in mucus clearance as a result of PEP treatment compared to a control session of coughing [32]. Bannier et al. performed hyperpolarized ^3^He MRI for the detection of peripheral airway obstruction [21]. Although spirometry indicated normal lung function, ventilation defects were found in all subjects. After physiotherapy, heterogeneous changes were observed in the distribution and extent of ventilation defects, but the mean values did not change significantly.

### Results: long-term effects

Three studies made chest X-rays at baseline and after a long-term intervention period [41, 46, 49]. Andréasson et al. and McIlwaine et al. (1997) scored the radiographs according to Brasfield’s scoring system [41, 49]. Andréasson et al. found no significant change after 2.5 years of daily exercise [49]. McIlwaine et al. (1997) compared two types of ACTs, conventional CPT and PEP, but no differences were found after one year [41]. Another study by McIlwaine et al. (2001) evaluated chest radiographs at the beginning and end of a one-year study as well, but the evaluation method was not specified by the authors [46]. No significant differences were found between the group using a PEP mask and the group using an OPEP device (Flutter).

### Discussion

Chest X-rays have been most frequently used to evaluate respiratory physiotherapy in children with chronic obstructive lung diseases. Chest X-rays are fast, easy to acquire and no voluntary effort is needed. Unfortunately, scoring systems were not able to detect any changes in disease progression or differences between treatment modalities in long-term trials with variable LOEs.

The potential of monitoring lung disease by MRI has increased over the past decade, since modern MRI techniques are able to provide information regarding ventilation and gas exchange, besides structural information [76]. One uncontrolled level 2b study included in this review applied hyperpolarized ^3^He MRI to visualize ventilation defects. Although no significant changes were found, the individual results indicated movement or relief of mucus plugging induced by physiotherapy [21]. The combination of structural and functional information acquired by MRI techniques could give more insight into the working mechanisms of different ACTs in future research.

Lastly, one study measured acute changes of airway clearance by RAT, but no improvement in mucus transport could be found [32]. Contrary to these results, studies including adult patients with CF were able to show an increased clearance attributable to physiotherapy [77]. Advantages of this technique are that it is an objective measure of mucus transport and it allows differentiation between different areas of the lungs. Unfortunately, this technique is highly expensive and requires radioisotopes, which are difficult to obtain.

## Disease exacerbation parameters

### Description

The section ‘disease exacerbation parameters’ summarizes parameters related to the worsening of disease, such as number or days of hospitalization, days of antibiotics, number of respiratory infections, etc. Pulmonary exacerbations are associated with a worse long-term prognosis and a decrease in health-related quality of life (QoL) in children with chronic obstructive lung diseases [78–80]. Hence, these parameters are an important aspect of the evaluation of long-term beneficial effects of ACTs in this population.

### Results: long-term effects

A total of ten studies included parameters related to the worsening of disease [39–43, 46, 48, 50, 52, 56], of which eight compared two different treatment regimens [39–41, 43, 46, 48, 50, 56]. Only two could find a significant difference, both of them performed by McIlwaine and colleagues [46, 50]. In those studies, PEP treatment resulted in fewer hospitalizations compared to OPEP [46], and in fewer antibiotic treatments compared to HFCWO during a one year intervention period [50]. The two level 2b RCTs recruited a sufficient number of patients, namely 40 and 107, but in both of them less than 85% of the participants completed the one-year intervention. Plebani et al. evaluated the efficacy of one year of daily physiotherapy treatments using a PEP-mask in a group of HIV-infected children with recurrent pulmonary infections [42]. They observed a reduction in the mean number of infections, antibiotic treatments and antibiotic days in comparison with the previous year without prophylactic physiotherapy. It should be noted that the latter study is considered as level 4 evidence. Ghasempour et al. matched a group of CF patients receiving PEP treatment with a group receiving no physiotherapy during a pulmonary exacerbation [52]. This level 1b RCT found a significantly lower rate of rehospitalization within the six months follow-up for the experimental group.

### Discussion

Airway clearance strategies aim to facilitate mucus transport and to reduce mucus stasis in the lungs to reduce pulmonary infections. However, the majority of the studies evaluating two types of ACTs did not find a significant difference between therapies [39–41, 43, 48, 56]. As most studies reported parameters regarding hospitalization, mainly severe exacerbations were evaluated. Mild community-managed PEs could also be considered by assessing specific signs/symptoms related to disease worsening and the need for additional treatment, which might increase the sensitivity to detect any differences between types of therapy. In contrast to studies evaluating different ACT, both studies comparing their results to a control period or group were able to demonstrate beneficial effects of ACTs on this type of parameters [42, 52]. An advantage of these parameters is that no complex testing material is required to collect this information. On the other hand, the parameters are influenced by the treatment strategy and judgement of the physician, which can differ by country.

## Patient-reported outcomes

### Description

The impact on health-related QoL and individual preference are important but often overlooked aspects of airway clearance [81]. Different elements can be evaluated, such as symptom perception, daily activities, patient satisfaction, etc. For CF, the Cystic Fibrosis Questionnaire – Revised (CFQ-R) is the most well-known validated and reliable questionnaire available for both adults and children, which assesses several of the elements described above in five separate domains [82]. Besides questionnaires, a Likert scale or a visual analogue scale (VAS) can be used to measure certain features, such as patient satisfaction or dyspnea. PROs can be reliably and consistently reported by children from the age of 8 years. In younger children information can be acquired from a parent/caregiver, but these responses cannot be considered interchangeable or combined in data analysis [83].

### Results: short-term effects within 24 h

Three articles included a patient-reported outcome (PRO) measure to evaluate one treatment session, of which only the study by Reix et al. performed a statistical analysis of the results [16, 22, 36]. Reix et al. compared exercise with expiratory maneuvers to a session of ACBT in a randomized cross-over trial [22]. They asked patients to rate the quality of each session on a 1 to 5 Likert scale, and the satisfaction of each treatment on a 0 to 100 VAS. Only the difference in perceived satisfaction between groups reached statistical significance in favor of the exercise session. The other two studies by Fauroux et al. and Phillips et al. reported marked differences in patient perception between types of treatment, which were related to comfort, effort, breathlessness during treatment and/or the ease to clear secretions.

### Results: long-term effects

A total of five studies assessed patient preference and/or health-related QoL after an intervention period ranging from five days to one year [40, 41, 48, 50, 51]. Of these five studies three were performed by McIlwaine and colleagues. Although no statistical analysis was performed in their level 1b studies published in 1997 and 2010, a unanimous preference was reported for PEP and AD over CPT, respectively [41, 48]. In a third level 2b study conducted in 2013, the CFQ-R was used to assess QoL and a 1 to 5 points VAS to measure differences in satisfaction between the interventions [50]. The CFQ-R scores did not significantly differ over time nor between PEP and HFCWO therapy. Also, no significant differences were observed for comfort and independence. Only flexibility scored higher in the PEP group, which was related to the flexibility in where they could perform their ACT. Homnick et al. reported patient preference on a 0 to 10 scale in only the experimental group receiving IPV [40]. All patients reported a high satisfaction using the IPV device and the majority of the patients would continue to use IPV if given the opportunity. In a 5-day clinical trial, Gokdemir et al. found HFCWO was rated significantly more comfortable than conventional CPT rated on a 5-point scale [51].

### Discussion

In general, PROs are included in a number of studies to capture the subjective effect of the different treatments. They can either consist of validated questionnaires or specifically developed questions using a Likert scale or VAS. In contrast to objective parameters, it seems that the subjective perception of the patient often shows differences between types of ACT. Unfortunately, only three studies included in this review performed statistical analyses to support their findings [22, 50, 51]. It is not always clear whether these differences can be linked to a change in a clinical parameter, such as in lung function or sputum expectoration. It is possible that the patient is biased. Another possible explanation might be that changes are missed by conventional outcome parameters. Therefore, it is important to incorporate these PROs in clinical practice as well as in new study designs. By considering the impact of the treatment on QoL, personal health beliefs and patient preference, a patient-centered approach is promoted, which will also optimize the adherence to the therapy [84].

## Other

Clinical scoring systems were used in a total of six studies to assess the effects of a long-term intervention [43, 46–49, 56]. The most frequently used and most well-known scoring system to evaluate disease progression in CF is the Shwachman score developed in 1958. No differences between types of therapy were noted in the included studies [46, 48, 56]. Only one study reported a significant decline after three years compared to baseline in the group performing FET only, while the clinical status remained stable in group receiving CPT [56]. By contrast, the Huang score was able to find significant differences between treatment regimens in both studies performed by McIlwaine et al. [46, 48]. The latter score, however, has not been fully validated in children [85]. Two other scoring systems, the Modified Case Western and the Clinical Asthma Score, were based on methods applied by other researchers [86–88] and were adapted according to the relevance for the study. To our knowledge, the validity and reliability of these lists have not been assessed. In general, clinical scoring lists can be valuable tools to describe different aspects contributing to the current clinical status. However, most scoring systems are considered outdated, because of the poor intra- and interobserver agreement, the lack of standardization and sensitivity. To date, computerized technology in data analysis and more objective outcome measures combined with measurements assessing quality of life obviate the use of antiquated clinical scoring systems [85].

Airway pathogens, especially *Pseudomonas aeruginosa*, are known to be associated with adverse clinical outcomes in patients with CF [89]. It is, however, unclear whether adequate mobilization of secretions influences the bacterial load. Only McIlwaine et al. obtained sputum samples every three months for bacteriologic culture. No significant changes were found over one year in either the PEP group or the OPEP group [46]. A pilot study by Dingemans et al., studied the influence of shear stress induced by IPV on the bacterial load and gene expression of *P. aeruginosa* in adults with CF [90]. They concluded that IPV at high frequency could potentially alter the behavior of *P. aeruginosa*, but this should be verified in a larger group of patients.

Apart from a study published in 1962 by Denton et al., no studies were found performing lung auscultation to evaluate ACTs in children with chronic obstructive lung diseases. Denton et al. reported that normal breathing sounds improved and the presence of rhonchi decreased in the majority of patients after one session of conventional CPT [12]. However, no statistical analysis was performed to endorse these results. Computer Aided Lung Sound Analysis (CALSA) has earlier been proposed to be a convenient outcome measure for respiratory physiotherapy [5]. Since previous studies including adult patients indicated that CALSA shows potential to be a sensitive measure for the detection of local changes in the airways after airway clearance [91–94]. Future research is required to assess the abilities of CALSA to quantify treatment effects of ACTs in the pediatric population.

## General discussion

The aim of this systematic review is to provide an overview of all outcome measures used in previous research to assess the treatment effects of ACTs in children with chronic obstructive lung diseases. No ideal method could be identified, because all types of outcome measures have their benefits and disadvantages. A summary of the most important considerations of the discussed outcome measures is provided in Table 2. A combination of different techniques is advised to gain a better understanding and to identify the potential effects of ACTs. The choice of outcomes will depend on the study design, population and objectives of the study. Methods related to mucus shifting, the expectoration of mucus and regional changes in the respiratory system are needed to gain more insight into the mode of action of the treatment. These methods will especially be of interest for studies focusing on the acute effects. To measure the impact on the clinical benefit, greater emphasis is placed on parameters related to disease severity and health-related QoL.

**Table 2:** Key considerations

Outcome measure	Indications, advantages	Limitations
Pulmonary function	- Suitable for long-term studies. - Conventional PFT (i.e. spirometry, body plethysmography): valid measures, reference equations and extensive guidelines available.	- Inappropriate to detect acute changes related to ACTs. - Pulmonary function is measured as a single unit (‘black box’ principle). No regional abnormalities or changes can be detected. - Conventional PFT: insensitive to mild lung disease. - Age appropriate approach required. - The potential of PFT in infants and preschoolers remains unclear.
Expectorated sputum	- Sputum quantity gives an impression of mucus transport in short-term studies.	- Sputum quantity: inaccurate, unreliable, unsuitable for uncooperative children.
Oxygen measurements	- Provides information about the presence of a ventilation-perfusion mismatch. - ABG analysis is the ‘gold standard’ method to measure blood oxygenation status. - Pulse oximetry is suitable for continuous monitoring and is simple to perform.	- ABG analysis requires invasive sampling. - Pulse oximetry is too imprecise for research purposes to detect small changes. - Oxygenation as an outcome is not suitable for children with mild lung disease in stable conditions.
Exercise capacity	- Adequate for long-term studies focused on pulmonary rehabilitation.	- Exercise capacity cannot be measured in children <6y. - Inappropriate to evaluate short-term effects of airway clearance. - Inadequate to measure solely the effects of airway clearance.
Imaging	- Detailed regional information of the lungs. - Hyperpolarized MRI is sensitive to changes in ventilation distribution. - RAT technique is the most direct technique to quantify acute changes in mucus transport.	- Chest X-rays lack sufficient sensitivity. - CT imaging is associated with radiation exposure. - Subjectivity of scoring methods. - Limited availability and high cost of most techniques.
Disease exacerbation parameters	- Demonstrate the impact on pulmonary exacerbations, which is a direct clinical endpoint. - No complex testing material is required to achieve this information.	- Only relevant for long-term studies.
Patient-reported outcomes	- The inclusion of PROs promotes a patient-centered model of care. - The perception and preference of the patient will influence adherence to the therapy, which emphasizes the importance of PROs for the evaluation of ACTs.	- There are a large number of PROs available, but not all PROs are validated and therefore results should be interpreted with caution. - High risk of bias if subjects are not blinded to the therapy.

In pediatrics, especially in children younger than six years old, the choice of technique is more challenging due to the lack of cooperation and fully understanding instructions. In addition, the majority of children with chronic obstructive lung diseases, such as CF and PCD, will have only mild structural lung damage at this young age. Other than PFTs, no other type of outcome has been used to evaluate airway clearance in children younger than 6 years old. Consequently, there are still lots of opportunities for future research in this area.

In general, this review summarizes a heterogeneous group of studies performed over several decades. There was a wide variety in the application of certain outcome measures, study designs, types of therapy, etc. Overall, a small number of participants was recruited, with only 13 out of 40 studies including over 20 participants per intervention group [12, 14, 22, 23, 29, 39, 44, 46, 50–52, 56, 60]. Hence, it is not our intention to make any statements regarding the effectivity of ACTs, but to review the applicability of different outcome measures in children with chronic obstructive lung diseases. Eighty-eight percent of all studies examined children with CF. The recommendations made are, therefore, primarily directed to this respiratory disease. Regardless of similarities with other chronic obstructive lung diseases in pediatrics, caution is advised when extrapolating conclusions. Furthermore, the majority of the studies scored poorly on criteria regarding blinding and no placebo therapy could be offered, but these risks are inherent to physiotherapy studies.

For future research, we would like to point out two main areas of interest. The first is the evaluation of outcome measures suitable for infants and preschoolers. Several techniques have been proposed to be suitable outcome measures for research in this age group, such as MBW [95] and hyperpolarized MRI [96], but neither of them have been applied to measure treatment effects of respiratory physiotherapy. Important considerations for children younger than six years of age include feasibility of the procedure and sufficient sensitivity to measure treatment effects in patients with mild respiratory disease. A second area of interest is the evaluation of techniques assessing local changes in the airways related to airflow and resistance. Such techniques could provide valuable information about the physiological mechanisms of ACTs, which in turn could identify differences between types of therapy. As mentioned previously, computerized lung sound monitoring could potentially be a sensitive measure to quantify regional changes in the airways due to mucus displacement and/or improving local ventilation. Also imaging techniques combining structural and functional information could increase current knowledge regarding the effects of ACTs.

To conclude, there is an urgent need for appropriate tools to assess the effectiveness of an airway clearance regimen, since no ‘gold standard’ is available. This review highlighted the main advantages and limitations of current techniques and opportunities for future research. Researchers and respiratory physiotherapists will need to consider the provided recommendations depending on the study design, such as the main objective, the intervention period and population.

## Supplementary information

**Additional file 1: Table S1.** PEDro scale. **Table S2.** Overview of studies evaluating pulmonary function. **Table S3.** Overview of studies evaluating expectorated sputum. **Table S4.** Overview of studies evaluating oxygenation. **Table S5.** Overview of studies evaluating exercise capacity. **Table S6.** Overview of studies performing imaging techniques. **Table S7.** Overview of studies evaluating disease exacerbation parameters. **Table S8.** Overview studies evaluating patient-reported outcomes.

## Data Availability

Data sharing is not applicable to this article as no datasets were generated or analyzed during the current study.

## Abbreviations

6MWT: Six minute walk test
ABG: Arterial blood gas
ACBT: Active cycle of breathing technique
ACT: Airway clearance technique
AD: Autogenic drainage
CALSA: Computer aided lung sound analysis
CF: Cystic fibrosis
CFQ-R: Cystic fibrosis questionnaire – revised
COPD: Chronic obstructive pulmonary disease
CPET: Cardiopulmonary exercise testing
CPT: Chest physical therapy
CT: Computed tomography
EMA: European Medicines Agency
FDA: North American Food and Drug Association
FEF_25–75%_: Forced expiratory flow between 25 and 75% of the forced vital capacity
FET: Forced expiration technique
FEV_1_: Forced expiratory volume in one second
FRC: Functional residual capacity
FVC: Forced vital capacity
HFCWO: High-frequency chest wall oscillation
HIV: Human immunodeficiency virus
IPV: Intrapulmonary percussive ventilation
MBW: Multiple-breath washout
MRI: Magnetic resonance imaging
OPEP: Oscillatory expiratory pressure
PCD: Primary ciliary dyskinesia
PEF: Peak expiratory flow
PEP: Positive expiratory pressure
PFT: Pulmonary function test
PRISMA: Preferred Reporting Items for Systematic Reviews and Meta-analyses
PRO: Patient-reported outcome
PSV: Pressure support ventilation
QoL: Quality of life
RAT: Radioactive aerosol tracer
RCT: Randomized controlled trial
SpO_2_: Saturation of peripheral oxygen
VAS: Visual analogue scale
