# Supplementary material for: Outcome measures for airway clearance techniques in children with chronic obstructive lung diseases: a systematic review
Source: Respir Res. 2020 Aug 17;21:217. doi: 10.1186/s12931-020-01484-z (PMC7433087; doi:10.1186/s12931-020-01484-z)
Supplement: Supplementary file 1 — Additional file 1: Table S1. PEDro scale. Table S2. Overview of studies evaluating pulmonary function. Table S3. Overview of studies evaluating expectorated sputum. Table S4. Overview of studies evaluating oxygenation. Table S5. Overview of studies evaluating exercise capacity. Table S6. Overview of studies performing imaging techniques. Table S7. Overview of studies evaluating disease exacerbation parameters. Table S8. Overview studies evaluating patient-reported outcomes. [file 12931_2020_1484_MOESM1_ESM.docx]

**Table S1.** PEDro scale.

| **Author (year)** | **1** | **2** | **3** | **4** | **5** | **6** | **7** | **8** | **9** | **10** | **11** | **Total score** |
| --- | --- | --- | --- | --- | --- | --- | --- | --- | --- | --- | --- | --- |
| Denton 1962 | 0 | 0 | 0 | 0 | 0 | 0 | 0 | 0 | 0 | 0 | 0 | **0** |
| Maxwell 1979 | 1 | 1 | 0 | 1 | 0 | 0 | 0 | 0 | 0 | 0 | 0 | **2** |
| Weller 1980 | 0 | 0 | 0 | 0 | 0 | 0 | 0 | 0 | 0 | 0 | 1 | **1** |
| Zach 1982 | 0 | 0 | 0 | 0 | 0 | 0 | 0 | 0 | 0 | 0 | 1 | **1** |
| Desmond 1983 | 1 | 0 | 0 | 1 | 0 | 0 | 1 | 0 | 0 | 1 | 0 | **3** |
| De Boeck 1984 | 0 | 1 | 0 | 1 | 0 | 0 | 1 | 0 | 0 | 1 | 1 | **5** |
| Andreasson 1987 | 0 | 0 | 0 | 0 | 0 | 0 | 0 | 0 | 1 | 0 | 1 | **2** |
| Van Asperen 1987 | 1 | 1 | 0 | 1 | 0 | 0 | 0 | 0 | 0 | 1 | 1 | **4** |
| Bain 1988 | 1 | 1 | 0 | 1 | 0 | 0 | 0 | 0 | 0 | 1 | 1 | **4** |
| Reisman 1988 | 1 | 1 | 0 | 1 | 0 | 0 | 0 | 1 | 0 | 1 | 1 | **5** |
| Cerny 1989 | 1 | 1 | 0 | 0 | 0 | 0 | 0 | 1 | 0 | 1 | 1 | **4** |
| Maayan 1989 | 0 | 1 | 0 | 1 | 0 | 0 | 0 | 0 | 0 | 1 | 1 | **4** |
| Asher 1990 | 1 | 1 | 0 | 1 | 0 | 0 | 1 | 1 | 0 | 1 | 1 | **6** |
| Oberwaldner 1991 | 0 | 0 | 0 | 0 | 0 | 0 | 0 | 0 | 0 | 0 | 1 | **1** |
| Steen 1991 | 0 | 1 | 0 | 1 | 0 | 0 | 0 | 1 | 0 | 1 | 1 | **5** |
| Van der Schans 1991 | 0 | 1 | 0 | 1 | 0 | 0 | 0 | 0 | 0 | 1 | 1 | **4** |
| Pfleger 1992 | 1 | 1 | 0 | 1 | 0 | 0 | 1 | 1 | 0 | 1 | 1 | **6** |
| Bauer 1994 | 1 | 1 | 0 | 1 | 0 | 0 | 0 | 1 | 0 | 1 | 1 | **5** |
| Natale 1994 | 1 | 1 | 0 | 1 | 0 | 0 | 1 | 1 | 0 | 1 | 1 | **6** |
| Homnick 1995 | 1 | 1 | 0 | 1 | 0 | 0 | 0 | 0 | 0 | 1 | 1 | **4** |
| Mcllwaine 1997 | 0 | 1 | 0 | 1 | 0 | 0 | 1 | 1 | 0 | 1 | 1 | **6** |
| Plebani 1997 | 1 | 0 | 0 | 0 | 0 | 0 | 0 | 1 | 1 | 0 | 1 | **3** |
| Homnick 1998 | 1 | 0 | 0 | 1 | 0 | 0 | 0 | 0 | 0 | 1 | 1 | **3** |
| Newhouse 1998 | 1 | 1 | 0 | 1 | 0 | 0 | 1 | 0 | 0 | 1 | 1 | **5** |
| Van Winden 1998 | 0 | 1 | 0 | 1 | 0 | 0 | 0 | 1 | 1 | 1 | 1 | **6** |

**Table S1.** Continued

| **Author (year)** | **1** | **2** | **3** | **4** | **5** | **6** | **7** | **8** | **9** | **10** | **11** | **Total score** |
| --- | --- | --- | --- | --- | --- | --- | --- | --- | --- | --- | --- | --- |
| Fauroux 1999 | 0 | 1 | 0 | 1 | 0 | 0 | 0 | 1 | 1 | 1 | 1 | **6** |
| Gondor 1999 | 1 | 1 | 0 | 1 | 0 | 0 | 1 | 1 | 0 | 1 | 1 | **6** |
| Williams 2000 | 1 | 1 | 0 | 0 | 0 | 0 | 0 | 0 | 0 | 1 | 1 | **3** |
| Mcllwaine 2001 | 1 | 1 | 0 | 1 | 0 | 0 | 1 | 0 | 0 | 1 | 1 | **5** |
| Williams 2001 | 1 | 1 | 0 | 1 | 0 | 0 | 0 | 0 | 0 | 1 | 1 | **4** |
| Samransamruajkit 2003 | 1 | 1 | 1 | 0 | 0 | 0 | 0 | 1 | 0 | 1 | 1 | **5** |
| Marks 2004 | 1 | 1 | 0 | 1 | 0 | 0 | 1 | 1 | 0 | 1 | 1 | **6** |
| Phillips 2004 | 1 | 1 | 1 | 1 | 0 | 0 | 1 | 0 | 0 | 1 | 1 | **6** |
| Darbee 2005 | 1 | 0 | 0 | 1 | 0 | 0 | 0 | 1 | 1 | 1 | 1 | **5** |
| Lagerkvist 2006 | 0 | 1 | 0 | 1 | 0 | 0 | 0 | 1 | 0 | 1 | 1 | **5** |
| Hristara-Papadopoulou 2007 | 0 | 1 | 0 | 0 | 0 | 0 | 0 | 0 | 0 | 1 | 0 | **2** |
| Indinnimeo 2007 | 1 | 1 | 0 | 1 | 0 | 0 | 1 | 0 | 0 | 1 | 1 | **5** |
| Tannenbaum 2007 | 1 | 1 | 1 | 1 | 1 | 0 | 1 | 1 | 1 | 1 | 1 | **9** |
| Didario 2009 | 1 | 1 | 0 | 1 | 0 | 0 | 0 | 1 | 0 | 1 | 0 | **4** |
| Bannier 2010 | 0 | 0 | 0 | 0 | 0 | 0 | 0 | 1 | 1 | 0 | 1 | **3** |
| Mcllwaine 2010 | 0 | 1 | 0 | 1 | 0 | 0 | 1 | 1 | 0 | 1 | 1 | **6** |
| Reix 2012 | 1 | 1 | 1 | 1 | 0 | 0 | 0 | 1 | 1 | 1 | 1 | **7** |
| Abbas 2013 | 0 | 0 | 0 | 0 | 0 | 0 | 0 | 1 | 0 | 1 | 1 | **3** |
| Mcllwaine 2013 | 1 | 1 | 0 | 1 | 0 | 0 | 1 | 0 | 1 | 1 | 1 | **6** |
| Gokdemir 2014 | 1 | 1 | 0 | 1 | 0 | 0 | 1 | 0 | 0 | 1 | 1 | **5** |
| Rodriguez 2014 | 1 | 0 | 0 | 0 | 0 | 0 | 0 | 1 | 1 | 0 | 1 | **3** |
| Voldby 2018 | 1 | 0 | 0 | 0 | 0 | 0 | 0 | 1 | 0 | 0 | 1 | **2** |
| Ghasempour 2019 | 0 | 1 | 0 | 1 | 0 | 0 | 0 | 1 | 1 | 1 | 1 | **6** |
| Vendrusculo 2019 | 1 | 1 | 1 | 1 | 0 | 0 | 1 | 0 | 0 | 1 | 1 | **6** |

Criteria: 1, eligibility criteria; 2, randomisation; 3, concealed allocation; 4, similarity of groups at baseline; 5, blinding subjects; 6, blinding therapists, 7, blinding assessors; 8, >85% follow-up; 9, intention-to-treat analysis; 10, between-group statistical comparison; 11, point/variability measures. Score: 0, not present or not reported; 1, present. Total score: sum criteria 2-11.

**Table S2.** Overview of studies evaluating pulmonary function.

| **Author (year)** | **ACT** | **Spirometry** | | | | | | **Body plethysmography** | | | | | |
| --- | --- | --- | --- | --- | --- | --- | --- | --- | --- | --- | --- | --- | --- |
|  |  | vs. baseline | | vs. control | | vs. ACT | | vs. baseline | | vs. control | | vs. ACT | |
|  |  | S | NS | S | NS | S | NS | S | NS | S | NS | S | NS |
| ***Short-term*** | | | | | | | | | | | | | |
| Denton 1962 | CPT | *no statistical analysis* | | | | | |  |  |  |  |  |  |
| Maxwell 1979 | CPT |  |  |  |  |  | ● |  |  |  |  |  |  |
| Weller 1980 | CPT | ↑ |  | ↑ |  |  |  |  |  |  |  |  |  |
| De Boeck 1984 | CPT | ↑ |  |  |  |  | ● |  | ● |  |  |  | ● |
|  | Directed coughing |  | ● |  |  |  | ● |  | ● |  |  |  | ● |
| Van der Schans 1991 | low-PEP |  | ● |  |  |  |  |  | ● |  |  |  |  |
|  | high-PEP |  | ● |  |  |  |  |  | ● |  |  |  |  |
| Pfleger 1992 | PEP | ↑ |  |  |  |  | ● | ↑ |  |  |  |  | ● |
|  | AD | ↑ |  |  |  |  | ● |  | ● |  |  |  | ● |
|  | PEP + AD | ↑ |  |  |  |  | ● | ↑ |  |  |  |  | ● |
|  | AD + PEP |  | ● |  |  |  | ● |  | ● |  |  |  | ● |
| Natale 1994 | IPV |  |  |  |  |  | ● |  |  |  |  |  |  |
|  | CPT |  |  |  |  |  | ● |  |  |  |  |  |  |
| Newhouse 1998 | CPT |  | ● |  |  |  | ● | ● |  |  |  |  | ● |
|  | OPEP | ↑ |  |  |  |  | ● | ● |  |  |  |  | ● |
|  | IPV | ↑ |  |  |  |  | ● | ● |  |  |  |  | ● |
| Fauroux 1999 | FET | ↓ |  |  |  |  |  |  |  |  |  |  |  |
|  | FET + NIV |  | ● |  |  |  |  |  |  |  |  |  |  |
| Williams 2000 | ACBT + CPT | ↓ |  |  |  | ↑ |  |  |  |  |  |  |  |
|  | ACBT | ↓ |  |  |  | ↓ |  |  |  |  |  |  |  |
| Williams 2001 | ACBT + CPT |  | ● |  |  |  | ● | ● |  |  |  |  | ● |
|  | ACBT |  | ● |  |  |  | ● | ● |  |  |  |  | ● |
| Marks 2004 | CPT |  | ● |  |  |  | ● | ● |  |  |  |  | ● |
|  | IPV |  | ● |  |  |  | ● | ● |  |  |  |  | ● |
| Phillips 2004 | HFCWO |  | ● |  |  |  |  |  |  |  |  |  |  |
|  | ACBT | ↑ |  |  |  |  |  |  |  |  |  |  |  |
| Darbee 2005 | HFCWO | ↑ |  |  |  |  | ● |  |  |  |  |  |  |
|  | PEP | ↑ |  |  |  |  | ● |  |  |  |  |  |  |
| Lagerkvist 2006 | PEP |  | ● |  |  |  | ● |  |  |  |  |  |  |
|  | OPEP |  | ● |  |  |  | ● |  |  |  |  |  |  |
| Tannenbaum 2007 | CPT |  | ● |  | ● |  |  |  |  |  |  |  |  |
| Bannier 2010 | breathing exercises | ↓ |  |  |  |  |  |  |  |  |  |  |  |
| Reix 2012 | Exercise |  |  |  |  | ↑ |  |  |  |  |  |  |  |
|  | ACBT |  |  |  |  | ↓ |  |  |  |  |  |  |  |
| Abbas 2013 | PEP/OPEP |  | ● |  |  |  |  |  |  |  |  |  |  |
| Hortal 2014 | AD + PEP |  | ● |  |  |  |  |  |  |  |  |  |  |
| Voldby 2018 | Exercise + PEP +FET |  | ● |  |  |  |  |  |  |  |  |  |  |
| Vendrusculo 2019 | AD + PEP |  |  |  | ● |  |  |  |  | ↑ |  |  |  |

**Table S2.** Continued.

| **Author (year)** | **ACT** | **Spirometry** | | | | | | **Body plethysmography** | | | | | |
| --- | --- | --- | --- | --- | --- | --- | --- | --- | --- | --- | --- | --- | --- |
|  |  | vs. baseline | | vs. control | | vs. ACT | | vs. baseline | | vs. control | | vs. ACT | |
|  |  | S | NS | S | NS | S | NS | S | NS | S | NS | S | NS |
| ***Long-term*** | | | | | | | | | | | | | |
| Zach 1982 | Exercise | ↑ |  |  |  |  |  |  |  |  |  |  |  |
|  | CPT | ↓ |  |  |  |  |  |  |  |  |  |  |  |
| Desmond 1983 | CPT |  | ● | ↑ |  |  |  |  |  |  |  |  |  |
| Andreasson 1987 | Exercise |  | ● |  |  |  |  |  |  |  |  |  |  |
| Van Asperen 1987 | CPT |  | ● |  |  |  | ● |  |  |  |  |  |  |
|  | PEP |  | ● |  |  |  | ● |  |  |  |  |  |  |
| Bain 1988 | CPT | ↑ |  |  |  |  | ● | ↑ |  |  |  |  | ● |
|  | Directed coughing | ↑ |  |  |  |  | ● | ↑ |  |  |  |  | ● |
| Reisman 1988 | CPT | ↓ |  |  |  | ↑ |  |  |  |  |  |  |  |
|  | FET | ↓ |  |  |  | ↓ |  |  |  |  |  |  |  |
| Cerny 1989 | CPT | ↑ |  |  |  |  | ● |  | ● |  |  |  | ● |
|  | CPT + exercise | ↑ |  |  |  |  | ● |  | ● |  |  |  | ● |
| Asher 1990 | CPT |  |  |  | ● |  |  |  |  |  | ● |  |  |
| Oberwaldner 1991 | PEP | ↑ |  |  |  |  |  | ↑ |  |  |  |  |  |
| Steen 1991 | CPT |  |  |  |  |  | ● |  |  |  |  |  |  |
|  | PEP + CPT |  |  |  |  |  | ● |  |  |  |  |  |  |
|  | PEP |  |  |  |  |  | ● |  |  |  |  |  |  |
|  | PEP + FET |  |  |  |  |  | ● |  |  |  |  |  |  |
| Bauer 1994 | CPT |  |  |  |  |  | ● |  |  |  |  |  |  |
| Homnick 1995 | CPT |  | ● |  |  |  | ● |  |  |  |  |  |  |
|  | IPV |  | ● |  |  |  | ● |  |  |  |  |  |  |
| McIlwaine 1997 | CPT |  |  |  |  | ↓ |  |  |  |  |  |  |  |
|  | PEP |  |  |  |  | ↑ |  |  |  |  |  |  |  |
| Plebani 1997 | PEP | *no statistical analysis* | | | | | |  |  |  |  |  |  |
| Homnick 1998 | CPT | ↑ |  |  |  |  | ● | ↑ |  |  |  |  | ● |
|  | OPEP | ↑ |  |  |  |  | ● | ↑ |  |  |  |  | ● |
| Van Winden 1998 | OPEP |  | ● |  |  |  | ● | ● |  |  |  |  | ● |
|  | PEP |  | ● |  |  |  | ● | ● |  |  |  |  | ● |
| Gondor 1999 | CPT | ↑ |  |  |  | ↓ |  |  |  |  |  |  |  |
|  | OPEP | ↑ |  |  |  | ↑ |  |  |  |  |  |  |  |
| McIlwaine 2001 | PEP |  | ● |  |  | ↑ |  |  |  |  |  |  |  |
|  | OPEP | ↓ |  |  |  | ↓ |  |  |  |  |  |  |  |
| Samransamruajkit 2003 | OPEP | ↑ |  | ↑ |  |  |  |  |  |  |  |  |  |
| Indinnimeo 2007 | CPT supervised | ↑ |  |  |  | ↑ |  | ↑ |  |  |  | ↑ |  |
|  | CPT unsupervised |  | ● |  |  | ↓ |  |  | ● |  |  | ↓ |  |
| McIlwaine 2010 | CPT |  |  |  |  |  | ● |  |  |  |  |  |  |
|  | AD |  |  |  |  |  | ● |  |  |  |  |  |  |
| McIlwaine 2013 | PEP | ↑ |  |  |  |  | ● |  |  |  |  |  |  |
|  | HFCWO | ↑ |  |  |  |  | ● |  |  |  |  |  |  |
| Gokdemir 2014 | CPT | ↑ |  |  |  |  | ● |  |  |  |  |  |  |
|  | HFCWO | ↑ |  |  |  |  | ● |  |  |  |  |  |  |
| Ghasempour 2019 | PEP | ↑ |  | ↑ |  |  |  |  |  |  |  |  |  |
| Abbreviations: ACBT, active cycle of breathing technique; ACT, airway clearance technique; AD, autogenic drainage; CPT, chest physical therapy; FET, forced expiration technique; HFCWO, high frequency chest wall oscillation; IPV, intrapulmonary percussive ventilation; NIV, non-invasive ventilation; NS, non-significant; (O)PEP (oscillatory) positive expiratory pressure; S, significant; ↑, improved/better result; ↓, deteriorated/worse result. | | | | | | | | | | | | | |

**Table S3.** Overview of studies evaluating expectorated sputum.

| **Author (year)** | **ACT** | **Expectorated sputum** | | | | | |
| --- | --- | --- | --- | --- | --- | --- | --- |
|  |  | vs. baseline | | vs. control | | vs. ACT | |
|  |  | S | NS | S | NS | S | NS |
| ***Short-term*** | | | | | | | |
| Denton 1962 | CPT | *no statistical analysis* | | | | | |
| Maxwell 1979 | CPT |  |  |  |  |  | ● |
| De Boeck 1984 | CPT |  |  |  |  |  | ● |
|  | Directed coughing |  |  |  |  |  | ● |
| Van Asperen 1987 | CPT |  |  |  |  |  | ● |
|  | PEP |  |  |  |  |  | ● |
| Steen 1991 | CPT |  |  |  |  |  | ● |
|  | PEP + CPT |  |  |  |  |  | ● |
|  | PEP |  |  |  |  |  | ● |
|  | PEP + FET |  |  |  |  |  | ● |
| Pfleger 1992 | PEP |  |  | ↑ |  | ↑ |  |
|  | AD |  |  | ↑ |  | ↓ |  |
|  | PEP + AD |  |  | ↑ |  | ↑ |  |
|  | AD + PEP |  |  | ↑ |  | ↑ |  |
| Natale 1994 | IPV |  |  |  |  |  | ● |
|  | CPT |  |  |  |  |  | ● |
| Newhouse 1998 | CPT |  |  |  |  |  | ● |
|  | OPEP |  |  |  |  |  | ● |
|  | IPV |  |  |  |  |  | ● |
| Fauroux 1999 | FET |  |  |  |  |  | ● |
|  | FET + NIV |  |  |  |  |  | ● |
| Williams 2000 | ACBT + CPT |  |  |  |  |  | ● |
|  | ACBT |  |  |  |  |  | ● |
| Marks 2004 | CPT |  |  |  |  |  | ● |
|  | IPV |  |  |  |  |  | ● |
| Phillips 2004 | HFCWO |  |  |  |  | ↓ |  |
|  | ACBT |  |  |  |  | ↑ |  |
| Hristrara-Papadopoulou 2007 | CPT |  |  |  |  | ↓ |  |
|  | CPT + ACBT |  |  |  |  | ↑ |  |
| Bannier 2010 | breathing exercises | *no statistical analysis* | | | | | |
| Reix 2012 | Exercise |  |  |  |  |  | ● |
|  | ACBT |  |  |  |  |  | ● |
| ***Long-term*** | | | | | | | |
| Desmond 1983 | CPT |  |  |  |  |  | ● |
| Bain 1988 | CPT | ↑ |  |  |  |  | ● |
|  | Directed coughing | ↑ |  |  |  |  | ● |
| Cerny 1989 | CPT |  |  |  |  |  | ● |
|  | CPT + Exercise |  |  |  |  |  | ● |
| Oberwaldner 1991 | PEP | *S correlated with PFT parameters* | | | | | |
| Abbreviations: ACBT, active cycle of breathing technique; ACT, airway clearance technique; AD, autogenic drainage; CPT, chest physical therapy; FET, forced expiration technique; HFCWO, high frequency chest wall oscillation; IPV, intrapulmonary percussive ventilation; NIV, non-invasive ventilation; NS, non-significant; (O)PEP (oscillatory) positive expiratory pressure; S, significant; ↑, improved/better result; ↓, deteriorated/worse result. | | | | | | | |

**Table S4.** Overview of studies evaluating oxygenation.

| **Author (year)** | **ACT** | **Oxygenation** | | | | | |
| --- | --- | --- | --- | --- | --- | --- | --- |
|  |  | vs. baseline | | vs. control | | vs. ACT | |
|  |  | S | NS | S | NS | S | NS |
| ***Short-term*** | | | | | | | |
| Fauroux 1999 | FET |  |  |  |  | ↓ |  |
|  | FET + NIV |  |  |  |  | ↑ |  |
| Darbee 2005 | HFCWO | ↓ |  |  |  | ↓ |  |
|  | PEP | ↑ |  |  |  | ↑ |  |
| Lagerkvist 2006 | PEP |  | ● |  |  | ↓ |  |
|  | OPEP | ↑ |  |  |  | ↑ |  |
| Didario 2009 | CPT |  | ● |  | ● |  |  |
| Gokdemir 2014 | CPT |  |  |  |  |  | ● |
|  | HFCWO |  |  |  |  |  | ● |
| ***Long-term*** | | | | | | | |
| Bain 1988 | CPT | ↑ |  |  |  |  | ● |
|  | Directed coughing | ↑ |  |  |  |  | ● |
| Samransamruajkit 2003 | OPEP |  |  |  | ● |  |  |
| Ghasempour 2019 | PEP |  | ↑ |  | ● |  |  |
| Abbreviations: ACT, airway clearance technique; CPT, chest physical therapy; FET, forced expiration technique; HFCWO, high frequency chest wall oscillation; NIV, non-invasive ventilation; NS, non-significant; (O)PEP (oscillatory) positive expiratory pressure; S, significant; ↑, improved/better result; ↓, deteriorated/worse result. | | | | | | | |

**Table S5.** Overview of studies evaluating exercise capacity.

| **Author (year)** | **ACT** | **Exercise capacity** | | | | | |
| --- | --- | --- | --- | --- | --- | --- | --- |
|  |  | vs. baseline | | vs. control | | vs. ACT | |
|  |  | S | NS | S | NS | S | NS |
| ***Short-term*** | | | | | | | |
| Vendrusculo 2019 | AD + PEP |  |  |  | ● |  |  |
| ***Long-term*** | | | | | | | |
| Andréasson 1987 | Exercise |  | ● |  |  |  |  |
| Reisman 1988 | CPT |  | ● |  |  |  | ● |
|  | FET |  | ● |  |  |  | ● |
| Cerny 1989 | CPT | ↑ |  |  |  |  | ● |
|  | CPT + exercise | ↑ |  |  |  |  | ● |
| Gondor 1999 | CPT | ↑ |  |  |  |  | ● |
|  | OPEP | ↑ |  |  |  |  | ● |
| Abbreviations: ACT, airway clearance technique; AD, autogenic drainage; CPT, chest physical therapy; FET, forced expiration technique; NS, non-significant; (O)PEP (oscillatory) positive expiratory pressure; S, significant; ↑, improved/better result; ↓, deteriorated/worse result. | | | | | | | |

**Table S6.** Overview of studies performing imaging techniques.

| **Author (year)** | **ACT** | **Imaging** | | | | | |
| --- | --- | --- | --- | --- | --- | --- | --- |
|  |  | vs. baseline | | vs. control | | vs. ACT | |
|  |  | S | NS | S | NS | S | NS |
| ***Short-term*** | | | | | | | |
| Van der Schans 1991 | low-PEP |  |  |  | ● |  | ● |
|  | high-PEP |  |  |  | ● |  | ● |
| Bannier 2010 | breathing exercises |  | ● |  |  |  |  |
| ***Long-term*** | | | | | | | |
| Andréasson 1987 | Exercise |  | ● |  |  |  |  |
| McIlwaine 1997 | CPT |  |  |  |  |  | ● |
|  | PEP |  |  |  |  |  | ● |
| McIlwaine 2001 | PEP |  |  |  |  |  | ● |
|  | OPEP |  |  |  |  |  | ● |
| Abbreviations: ACT, airway clearance technique; CPT, chest physical therapy; NS, non-significant; (O)PEP (oscillatory) positive expiratory pressure; S, significant; ↑, improved/better result; ↓, deteriorated/worse result. | | | | | | | |

**Table S7.** Overview of studies evaluating disease exacerbation parameters.

| **Author (year)** | **ACT** | **Disease exacerbation** | | | | | |
| --- | --- | --- | --- | --- | --- | --- | --- |
|  |  | vs. baseline | | vs. control | | vs. ACT | |
|  |  | S | NS | S | NS | S | NS |
| ***Long-term*** | | | | | | | |
| Reisman 1988 | CPT |  |  |  |  |  | ● |
|  | FET |  |  |  |  |  | ● |
| Bauer 1994 | CPT |  |  |  |  |  | ● |
| Homnick 1995 | CPT |  |  |  |  |  | ● |
|  | IPV |  |  |  |  |  | ● |
| McIlwaine 1997 | CPT |  |  |  |  |  | ● |
|  | PEP |  |  |  |  |  | ● |
| Plebani 1997 | PEP |  |  | ↑ |  |  |  |
| Homnick 1998 | CPT |  |  |  |  |  | ● |
|  | OPEP |  |  |  |  |  | ● |
| McIlwaine 2001 | PEP |  |  |  |  | ↑ |  |
|  | OPEP |  |  |  |  | ↓ |  |
| McIlwaine 2010 | CPT |  |  |  |  |  | ● |
|  | AD |  |  |  |  |  | ● |
| McIlwaine 2013 | PEP |  |  |  |  | ↑ |  |
|  | HFCWO |  |  |  |  | ↓ |  |
| Ghasempour 2019 | PEP |  |  | ↑ |  |  |  |
| Abbreviations: ACT, airway clearance technique; AD, autogenic drainage; CPT, chest physical therapy; FET, forced expiration technique; HFCWO, high frequency chest wall oscillation; IPV, intrapulmonary percussive ventilation; NS, non-significant; (O)PEP (oscillatory) positive expiratory pressure; S, significant; ↑, improved/better result; ↓, deteriorated/worse result. | | | | | | | |

**Table S8.** Overview studies evaluating patient-reported outcomes.

| **Author (year)** | **ACT** | **Patient-reported outcomes** | | | | | |
| --- | --- | --- | --- | --- | --- | --- | --- |
|  |  | vs. baseline | | vs. control | | vs. ACT | |
|  |  | S | NS | S | NS | S | NS |
| ***Short-term*** | | | | | | | |
| Fauroux 1999 | FET | *no statistical analysis* | | | | | |
|  | FET + NIV |  |  |  |  |  |  |
| Phillips 2004 | HFCWO | *no statistical analysis* | | | | | |
|  | ACBT |  |  |  |  |  |  |
| Reix 2012 | Exercise |  |  |  |  | ↑ |  |
|  | ACBT |  |  |  |  | ↓ |  |
| ***Long-term*** | | | | | | | |
| Homnick 1995 | CPT | *no statistical analysis* | | | | | |
|  | IPV |  |  |  |  |  |  |
| McIlwaine 1997 | CPT | *no statistical analysis* | | | | | |
|  | PEP |  |  |  |  |  |  |
| McIlwaine 2010 | CPT | *no statistical analysis* | | | | | |
|  | AD |  |  |  |  |  |  |
| McIlwaine 2013 | PEP |  |  |  |  | ↑ |  |
|  | HFCWO |  |  |  |  | ↓ |  |
| Gokdemir 2014 | CPT |  |  |  |  | ↓ |  |
|  | HFCWO |  |  |  |  | ↑ |  |
| Abbreviations: ACBT, active cycle of breathing technique; ACT, airway clearance technique; AD, autogenic drainage; CPT, chest physical therapy; FET, forced expiration technique; HFCWO, high frequency chest wall oscillation; IPV, intrapulmonary percussive ventilation; NIV, non-invasive ventilation; NS, non-significant; PEP, positive expiratory pressure; S, significant; ↑, improved/better result; ↓, deteriorated/worse result. | | | | | | | |
